# Unexpected Phenotype Reversion and Survival in a Zebrafish Model of Multiple Sulfatase Deficiency

**DOI:** 10.3389/fcell.2022.843079

**Published:** 2022-06-02

**Authors:** Angeleen Fleming, Low Zhe Xuan, Gentzane Sanchez-Elexpuru, Sarah V. Williams, Dylan Windell, Michael H. Gelb, Zackary M. Herbst, Lars Schlotawa, David C. Rubinsztein

**Affiliations:** ^1^ Department of Medical Genetics, Cambridge Institute for Medical Research, University of Cambridge, Cambridge, United Kingdom; ^2^ Department of Physiology, Development and Neuroscience, University of Cambridge, Cambridge, United Kingdom; ^3^ UK Dementia Research Institute, Cambridge Institute for Medical Research, University of Cambridge, Cambridge, United Kingdom; ^4^ Department of Chemistry, University of Washington, Seattle, WA, United States; ^5^ Department of Paediatrics and Adolescent Medicine, University Medical Centre Goettingen, Goettingen, Germany

**Keywords:** multiple sulfatase deficiency, formylglycine-generating enzyme, lysosome, zebrafish, SUMF1

## Abstract

Multiple sulfatase deficiency (MSD) is a rare recessively inherited Mendelian disorder that manifests with developmental delay, neurodegeneration, skeletal deformities, facial dysmorphism, congenital growth retardation, and other clinical signs. The disorder is caused by mutations in the *SUMF1* gene, which encodes the formylglycine-generating enzyme (FGE), and responsible for the activation of sulfatases. Mutations in *SUMF1* result in reduced or absent FGE function with consequent compromised activities of its client sulfatases. This leads to an accumulation of enzyme substrates, such as glycosaminoglycans and sulfolipids, within lysosomes and subsequently impaired lysosome function and cellular pathology. Currently, there are no disease modifying therapeutic options for MSD patients, hence the need for more suitable animal models to investigate the disorder. Here, we describe the characterisation of a *sumf1* null zebrafish model, which has negligible sulfatase activity. Our *sumf1* ^−/−^ zebrafish model successfully recapitulates the pathology of MSD such as cranial malformation, altered bone development, an enlarged population of microglia, and growth retardation during early development but lacks early lethality of mouse *Sumf1* ^−/−^ models. Notably, we provide evidence of recovery in MSD pathology during later developmental stages, resulting in homozygous mutants that are viable. Hence, our data suggest the possibility of a unique compensatory mechanism that allows the *sumf1* ^−/−^ null zebrafish to survive better than human MSD patients and mouse *Sumf1* ^−/−^ models.

## Introduction

Multiple sulfatase deficiency (MSD) is a rare, autosomal recessive disorder that encompasses the clinical characteristics of individual sulfatase deficiencies like mucopolysaccharidosis (MPS) and metachromatic leukodystrophy (MLD) (Schlotawa et al., 2020). Notably, MSD is part of a larger group of diseases classified as lysosomal storage disease (LSD). MSD is caused by the lack of post-translational modification of sulfatases due to a mutation in the Sulfatase Modifying Factor 1 (SUMF1) gene (Dierks et al., 2009). SUMF1 encodes the formylglycine-generating enzyme (FGE), which uniquely converts the cysteine residue into a C-formylglycine residue at the sulfatase catalytic site. This conversion into C-formylglycine is essential for the sulfatase enzymatic activity (Schmidt et al., 1995). Since there is no other enzyme which can perform this modification, MSD patients have reduced activities of sulfatases (Dierks et al., 2009).

Sulfatases play the unique biochemical role of hydrolysing sulfate ester bonds of glycosaminoglycans (GAGs), like heparan sulfate, dermatan sulfate, keratan sulfate, chondroitin sulfate, and hyaluronan (Sardiello et al., 2005), steroid hormones (e.g., dehydroepiandrosterone 3-sulfate) and sulfolipids (e.g., cerebroside-3-sulfate) (Dierks et al., 2009). GAGs are characterized by their long, unbranched polysaccharide chain with O-sulfate groups, and are derived from proteoglycan degradation. These highly negatively charged groups interact with the positive charge of surrounding protein ligands and signalling molecules (Holmborn et al., 2012). Proteoglycans, like heparan sulfate proteoglycans and chondroitin sulfate proteoglycans, consist of a core protein coupled with varying numbers, and types of GAGs. These proteoglycans are abundant in the extracellular matrix (ECM) and at the cell surface. During degradation, the proteoglycans are internalised by endocytosis and the protein core is proteolytically removed [reviewed in (Freeze et al., 2015)]. The remaining glycosaminoglycan chain is then completely degraded by a suite of lysosomal enzymes (Freeze et al., 2015). Unsurprisingly, inactive sulfatases caused by *SUMF1* mutations lead to GAG accumulation within the lysosome, which is associated with impaired lysosome function and apoptosis. Indeed, accumulation of glycosaminoglycan is observed in both mouse *Sumf1−/−* models and human MSD patients (Settembre et al., 2007; Kobolák et al., 2019). Storage of sulfolipids results from the lack of arylsulfatase A (ARSA) activity and causes demyelination of the central and peripheral nervous system in MLD and MSD (Diez-Roux and Ballabio, 2005). In addition, non-lysosomal sulfatases modulate heparan sulfate dependent cell signalling pathways (Dhoot et al., 2001; Lamanna et al., 2007).

MSD is estimated to occur in one in 500,000 individuals worldwide (Cappuccio et al., 2020) with more than 143 cases of MSD recorded in the scientific literature (Schlotawa et al., 2020). Most MSD patients carry hypomorphic mutations, resulting in some residual sulfatase activity, although there are rare cases where FGE function is completely abrogated due to nonsense mutations (Schlotawa et al., 2019). A complete loss of FGE function leads to neonatal onset of MSD pathology and early mortality (Schlotawa et al., 2019). Presently, there are no disease modifying therapeutic options for MSD. This makes the generation of suitable animal models important for the development of potential treatment strategies. A *Sumf1* knockout mouse model (*Sumf1−/−*) with no sulfatase activity has been established (Settembre et al., 2007). These mice display similar clinical and biochemical features to MSD patients, such as scoliosis, facial malformation, congenital growth retardation, reduced sulfatase activities, and GAG storage in tissues and organs. However, mouse models are limited by the stock maintenance cost and slower drug screening efficiency when compared to smaller animal models. Furthermore, since MSD usually has an early onset, this suggests that most, if not all, deformities occur during early development (Schlotawa et al., 2020). Unfortunately, the ability to study early development in mouse models is impeded by their internal embryonic development and lack of transparency. Despite the evolutionarily conserved nature of *sumf1*/Fge, *Drosophila* are not an ideal animal model since they lack vertebrate-specific organs and structures such as the skeleton and multichambered heart, both of which have distinctive defects in MSD.

**FIGURE 1:**
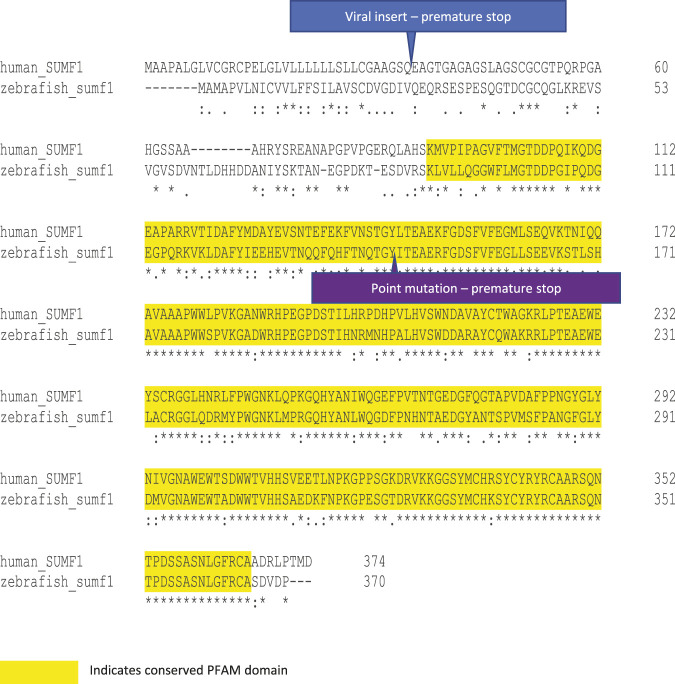
Protein alignment and mutation sites in human and zebrafish *SUMF1*/FGE. Human and zebrafish protein sequences share 59.94% sequence identify, increasing to 71.9% over the PFAM domain for FGE-sulfatase activity (yellow highlight). The viral insertion in the sumf1_la015919Tg line occurs in exon one and results in a premature stop codon (blue label). The point mutation in the sumf1_sa31531 line occurs in exon 2 and results in a nonsense mutation and premature stop codon (purple label).

The zebrafish has emerged as an attractive model for studying childhood onset disorders as they undergo external development and are transparent during early development, allowing early morphogenesis to be observed under a light microscope. Their relatively small size and transparency also lends itself to confocal microscopy to allow assessment of cells and tissues *in vivo*, using a range of fluorescent markers and reporters. Two zebrafish *sumf1* null lines have been identified from mutagenesis screens (Figure 1). The first line (sumf1_la015919Tg), was identified from a mutagenesis screen using random insertion of a murine leukaemia-based retrovirus (Wang et al., 2007). The retroviral insertion occurs in exon 1 resulting in a frameshift and stop codon that truncates the FGE protein enzyme rendering it inactive (Varshney et al., 2013). The second line (sumf1_sa31531) was generated by the Zebrafish Mutation Project using random ENU mutagenesis (Kettleborough et al., 2013). This line has a C > A mutation in exon 2, which also leads to a nonsense mutation. We confirmed an absence of sulfatase activity and an accumulation of GAGs in both lines but, surprisingly, found that *sumf1*−/− zebrafish were viable as adults unlike the *Sumf1* knockout mouse model, which display high mortality at 3 months (Settembre et al., 2007). This suggests the possibility that *sumf1* mutation affects zebrafish differently compared to its mammalian counterpart. We went on to characterise cartilage and bone development, microglial cell populations, and lysosome abundance and activity in the viral insertion *sumf1−/−* zebrafish model. Similar to what has been observed in mouse and human patients, the *sumf1−/−* zebrafish model displayed altered bone development, cranial shape deformation, and an increase in macrophage and microglia populations during early development, suggesting that our model recapitulates many clinical features seen in MSD human patients (Schlotawa et al., 1993). Surprisingly, these deformities show recovery at later stages indicating that possible compensatory mechanisms exist in zebrafish but not in their mammalian counterparts. However, there was no recovery of sulfatase activity and no evidence of a block in autophagic flux, indicating that zebrafish have alternative mechanisms to survive the accumulation of GAGs and sphingolipids. Taken together, this work demonstrates that *sumf1−/−* zebrafish recapitulate aspects of the early developmental defects associated with MSD and observed in *Sumf1* null mice. Strikingly however, the novel observation of disease recovery and viability in the zebrafish model suggests the presence of compensatory mechanisms which warrant further exploration.

## Materials and Methods

### Zebrafish Stock Maintenance and Embryo Production

All zebrafish procedures were performed in accordance with the United Kingdom Animals (Scientific Procedures) Act with appropriate Home Office Project and Personal animal licences and with local Ethics Committee approval. Experiments were performed in accordance with ARRIVE guidelines. Zebrafish were maintained on a 14 h light: 10 h dark cycle under standard conditions (Westerfield, 2000). Embryos were collected from natural spawnings, staged according to established criteria (Kimmel et al., 1995) and reared in embryo medium (5 mM NaCl, 0.17 mM KCl, 0.33 mM CaCl_2_, 0.33 mM Mg_2_SO_4_, 5 mM HEPES) at 28.5°C (hereafter referred to as E3M). The density of embryos was kept constant with embryo medium replenished daily. The sumf1_la015919Tg strain (ZFIN ID: ZDB-ALT-120806-11568) was used for the majority of experiments (hereafter referred to as *sumf1* ^*−/−*^), unless otherwise stated. The sumf1_sa 31531 was used in initial validation experiments to confirm that defects observed in the sumf1_la015919Tg strain were also observed in an independent line. The *sumf1* mutant lines were maintained on the Tuebingen Longfin (TL) background and this wild-type strain was used as the control line in all experiments. In all post-mortem analysis, zebrafish were culled by immersion in 1 mg/ml 3-amino benzoic acid ethyl ester (MS222, also known as tricaine) prior to tissue processing.

### Cartilage Staining

Zebrafish were fixed in 4% paraformaldehyde in phosphate-buffered saline (PBS), hereafter called PFA, overnight at 4°C. After three washes in dH_2_O, samples were stained with 0.1% Alcian Green overnight then differentiated in acid/alcohol (0.3% HCL, 70% Ethanol) and rehydrated by gradual replacement with dH_2_O. Subsequently, samples were digested using trypsin at room temperature for 10 min (for larvae) or 37°C overnight (for adult specimens) then bleached with 3% H_2_O_2_ in 1% KOH to remove all pigment, followed by 3 washes of 1% KOH. KOH was gradually replaced with glycerol until the samples had equilibrated and cleared.

### *In vivo* Bone Staining

Craniofacial and axial bones were detected by staining the zebrafish in E3M containing 0.1% Alizarin Red S (from a saturated solution in dH_2_0) and incubated in the dark at 28.5°C for 2–5 h depending on the age of the zebrafish. Fish were then washed three times in E3M, anaesthetised, and viewed using a dsRed filter set on a Leica M205A microscope.

### Wax Embedded Samples

Samples were fixed in Bouin’s fixative at RT for up to 6 months. The samples were dehydrated by immersing in the following series of solutions for 30 min (juvenile) and up to 60 min (adults): 70% Ethanol, 87% Ethanol, 95% Ethanol, 100% Ethanol, 100% Ethanol, 100% Ethanol/Histoclear (1:1), and Histoclear (2 washes in an oven set to 68.5°C). The histoclear was then gradually replaced with molten wax. The processed samples were placed in a sectioning mould and mounted using molten wax. Samples were sectioned at 10 μM using a Leica microtome (RM2125RT).

Transverse sections were used for the Alcian Blue staining. The sections were rehydrated through a graded alcohol series, stained in 1% Alcian Blue in 3% acetic acid (pH 2.5) for 30 min, rinsed and counterstained in 0.1% Nuclear Fast Red solution for 5 min. Sections were then dehydrated and mounted in Depex medium.

For Haematoxylin & Eosin (H&E) staining, the sections were rehydrated through a graded alcohol series, stained with Mayer´s Haematoxylin solution for 10 min, rinsed and dipped in Eosin (1% Eosin Y in dH_2_O) 12 times. Sections were then dehydrated and mounted in Depex medium.

### Protein Lysate Preparation

Larvae and adults were anaesthetized, excess liquid was removed, and lysis buffer containing 1% octylglucoside, complete protease inhibitor cocktail, and PhosSTOP™ tablets added. Samples were sonicated then centrifuged at 7000 rpm for 5 min at 4°C to obtain pure protein lysate. Total protein for each lysate was determined using a Pierce™ BCA Protein Assay Kit (Thermo Scientific™). Supernatants were diluted in 2 × Laemmli Buffer at a 1:1 dilution and boiled for 10 min before loading.

### Western Blotting

Samples were resolved by sodium dodecyl sulphate polyacrylamide gel electrophoresis using the BioRad mini-PROTEAN Tetra Electrophoresis System and transferred to the PVDF membranes. The membranes were blocked in 5% non-fat dry milk in PBST (PBS +0.1% Tween20) for 1 h at RT before staining in primary antibodies as denoted in Table 1. Membranes were incubated with primary antibodies overnight at 4°C (rocking). After three washes of PBST, membranes were incubated for one hour with secondary antibodies (Table 1) then washed three times in PBST. Immunoreactive bands were detected using ECL™ (GE Healthcare Bioscience) on a LI-COR Odyssey Fc Imager (LI-COR Biosciences) and processed with FIJI software (ImageJ).

**TABLE 1:** Western blotting reagents.

Antibody target	Primary/Secondary antibody	Concentration	Supplier
LC3 II (Rabbit)	Primary	1:500	Novus
Cathepsin D (Mouse)	Primary	1:500	Abcam
β-Actin (Mouse)	Primary	1:5000	Sigma-Aldrich
α-Tubulin (Mouse)	Primary	1:5000	Sigma-Aldrich
Anti-rabbit (Goat)	Secondary	1:5000	Dako
Anti-mouse (Goat)	Secondary	1:5000	Dako

### Wholemount Staining for Microglia/Macrophage

Staining was carried out as described (Astell and Sieger, 2017). Briefly, the paraformaldehyde-fixed samples were washed twice using PBStx (0.2% Triton X-100 in 0.01 M PBS) and blocked in 1% goat serum blocking buffer (1% normal goat serum, 1% DMSO, 1% BSA, and 0.7% Triton X-100 in 0.01M PBS) for 2 h. Subsequently, the samples were incubated with mouse anti-4C4 primary antibody (1:50; Abcam) overnight at 4°C. After two washes of PBStx, the samples were incubated with goat anti-mouse Alexafluor 488 in secondary antibody (1:1000; Thermo Fisher Scientific) in blocking buffer overnight at 4°C. After three washes of PBStx, the samples were stored in 70% glycerol at 4°C until the final mounting.

### Microscopy and Image Processing

Images for *in vivo* bone staining, cartilage staining, wholemount immunostaining were acquired using a fluorescence stereomicroscope (Leica; M205 FA) equipped with a Leica DFC7000T digital camera using the Leica Application Suite (LASX). Histological and immunostained sections were imaged using a Zeiss AxioPlan 2 Motorized Microscope equipped with a QImaging 2000R digital camera using QS imaging software. For wholemount immunostaining of macrophages and microglia, z-stacks were taken through the entire fish and LASX deconvolution was applied to the maximum intensity projection. For all images, the threshold was adjusted to 185 (min.) and 255 (max.) and particles within the z-stacks were counted.

### Lysotracker Staining

Larvae at 2 d.p.f, 5 d.p.f, and 10 d.p.f were incubated with 0.5% LysoTracker™ Red DND-99 (Invitrogen) in embryo medium for 45 min. 10 d.p.f larvae were treated from 1 d.p.f. with EM containing 0.03% phenylthiourea to prevent pigmentation. After staining, larvae were then anaesthetized, mounted in 1% low melting agarose in embryo medium and viewed using a Leica SP8 laser confocal microscope. Images were then processed with FIJI software (ImageJ).

### Sulfatase and Lysosomal Hydrolase Activity Assays

Lysis of fish larvae and tissues for activity assays was done as described above for western blot analysis. Instead of octylglucoside buffer lysis was done in presence of PBS (pH 7.4) and protease inhibitors (1% v/v, 1% Protease inhibitor mix, Roche, Mannheim, and Germany). Arylsulfatase A, N-acetylgalactosamine-6-sulfatase (GALNS) activity and N-sulfoglucosamine sulfohydrolase (SGSH) activity were determined following previously published protocols (Baum et al., 1959; Steckel et al., 1983; van Diggelen et al., 1990; Karpova et al., 1996). For β-galactosidase, lysates (2 µg) were diluted with substrate buffer (0.1 M citrate-phosphate pH 4.5, 2 mM 4-MU-β-D-galactopyranoside) Calbiochem Merck, Darmstadt, and Germany) to 40 µl final volume in wells of a black 96-well plate. A standard product dilution serious with 4-MU (Sigma Aldrich Merck, Darmstadt, Germany) diluted in H_2_O and 0.05 M Tris pH 8.0 was added. After incubation of 30 min at 37°C, the reaction was stopped with 150 µl stop buffer (0.17 M glycine-carbonate), and plates were centrifuged for 15 min at 1160 g. Readout was done using a fluorescent plate reader (Synergy Mx, BioTek, Winooski, United States) with excitation at 360 nm and emission at 460 nm. Activities were determined referring changes in OD or fluorescence respectively to total protein amounts and incubation time.

### GAG Analysis

Lysates of zebrafish larvae and adult brains were prepared as described for sulfatase and lysosomal hydrolase activity assays and analysed using the internal disaccharide method as described in (Herbst et al., 2020).

### Statistical Analysis

Statistical analysis was carried out using GraphPad Prism or Excel. Unpaired or Welch’s t-test were used for the analysis of two samples. One-way ANOVA followed by Tukeys multiple comparison test was used for comparing multiple samples.

## Results

### Sulfatase Activity and Survival

**FIGURE 2:**
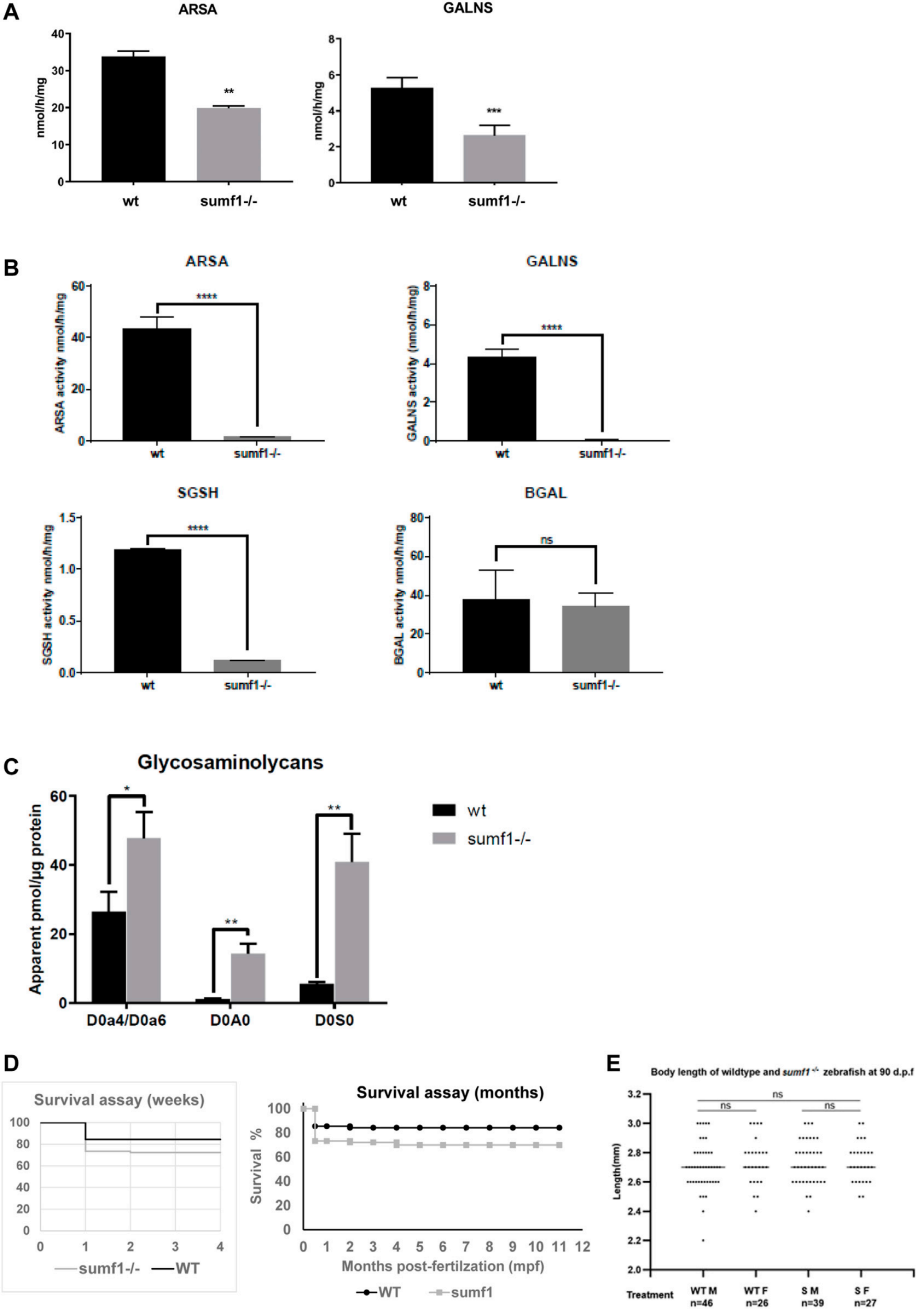
Sulfatase activity, GAG accumulation and viability of *sumf1* ^*−/−*^ zebrafish. **(A)** Activities of arylsulfatase A (ARSA) and N-acetylgalactosamine-6-sulfatase (GALNS), enzymes that are dependent on FGE function for their activation. Enzymatic activities were measured at 5 d.p.f. in larvae from in-crosses of *sumf1_LA* ^*+/-*^ adults. A significant reduction in sulfatase activity was confirmed in *sumf1* ^*−/−*^ larvae, indicating a loss of FGE function. **(B)** Since larvae from heterozygous females may retain maternal mRNA and/or protein, enzymatic activity assays were performed on *sumf1* ^*−/−*^ larvae from *sumf1* ^*−/−*^ parents and compared to wildtype larvae from a wildtype (TL) background. No or negligible levels of ARSA, GALNS, and N-Sulfoglucosamine sulfohydrolase (SGSH) activity could be detected in *sumf1* ^*−/−*^ larvae at 5 d.p.f. indicating an absence of FGE activity. Beta-galactosidase was included as a control as this enzyme is a lysosomal hydrolase which is not dependent on FGE. **(C)** Glycosaminoglycan levels were significantly elevated in *sumf1* ^*−/−*^ larvae at 5 d.p.f. relative to wildtype larvae of the same age. Chondroitin/Dermatan Sulfate (CS/DS) and Heparan Sulfate (HS) apparent levels were calculated using Chondrosine as an internal standard. D0a4: Chondroitin/Dermatan Sulfate Internal Disaccharide 4-sulfation (CS-A, DS) = unsaturated UA-GalNAc (4S); D0a6: Chondroitin Sulfate Internal Disaccharide 6-sulfation (CS-C) = unsaturated UA-GalNAc (6S); D0A0: Heparan Sulfate Internal Disaccharide n-sulfated = unsaturated UA-GlcNS; D0S0: Heparan Sulfate Internal Disaccharide no sulfation = unsaturated UA-GlcNAc. Apparent pmols were calculated using Chondrosine as an internal standard for mass spectrometry-based detection. **(D,E)** *sumf1* ^*−/−*^ zebrafish and wildtype fish were reared concomitantly and initially assessed weekly (for 1 month) and then monthly to assess survival. Although an initial drop in viability was observed in *sumf1* ^*−/−*^ larvae at 14 d.p.f., no further differences in viability were observed up to 12 months of age **(D)** and no differences in body length were observed in either sex at 30 d.p.f., suggesting that growth was normal **(E)**. For **(A–C)**, graphs show mean values (±SD) from at least 3 biological replicates. Statistical analysis was performed using two-tailed t-test. *p* > 0.05, *: *p* ≤ 0.05, **: *p* ≤ 0.01, and ***: *p* ≤ 0.001.

In-crosses of heterozygous fish for both the *sumf1_LA* and *sumf1_sa* lines produced viable offspring with the expected Mendelian distributions of wildtype, heterozygous and homozygous embryos and larvae. Embryos and larvae were observed daily up to 10 days post-fertilisation (d.p.f.), with no overt morphological differences observed between the genotypes. To confirm that the mutations in each line truly resulted a loss of FGE function, we analysed the activities of 2 different sulfatases that rely on FGE modification to become active (arylsulfatase A, ARSA and N-acetylgalactosamine-6-sulfatase, GALNS) in wildtype and homozygous siblings from in-crosses of *sumf1_LA* ^*+/-*^ adults at 5 d.p.f. A significant reduction in sulfatase activity was confirmed in *sumf1* ^*−/−*^ larvae, indicating a loss of FGE function (Figure 2A). Offspring from in-crosses of heterozygous fish were raised to adulthood and genotyped at 3 months post-fertilisation (m.p.f.). Normal Mendelian distributions of genotypes were observed for both *sumf1_LA* and *sumf1_sa* lines, hence longevity was analysed up to 2 years old and no differences were observed between genotypes. We therefore tested whether in-crosses of *sumf1* ^*−/−*^ adults produced viable offspring, since these females would produce eggs lacking maternally-deposited FGE protein and *sumf1* mRNA and therefore may be expected to have a more severe phenotype than those from *sumf1* ^*+/-*^ parents. All embryos and larvae from this cross were viable and with the expected Mendelian distributions of genotypes. To confirm that the offspring lacked FGE, we performed sulfatase activity assays on larvae at 5 d.p.f., comparing *sumf1* ^*−/−*^ larvae from *sumf1* ^*−/−*^ parents to wildtype larvae from a wildtype (TL) background. In *sumf1* ^*−/−*^ larvae, no or negligible levels of ARSA, GALNS and N-Sulfoglucosamine sulfohydrolase (SGSH) activity could be detected (Figure 2B), suggesting absent FGE activity. Beta-galactosidase was included as a control as this enzyme is a lysosomal hydrolase which is not dependent on FGE.

One would predict that an absence of sulfatase activity would result in an accumulation of glycosaminoglycans and, indeed, this was observed in *sumf1* ^*−/−*^ larvae relative to wildtype larvae of the same age (Figure 2C). Next, we examined the viability of *sumf1* ^*−/−*^ larvae from in-crosses of *sumf1* ^*−/−*^ adults and, although a drop in survival was observed at 14 d.p.f., we found no difference in long-term survival (Figure 2D), no difference in the size of *sumf1* ^*−/−*^ fish compared to wildtype fish reared in parallel (Figure 2E) and no overt histological differences were observed in muscle, brain or bone of *sumf1* ^*−/−*^ adults at 12 months old (Supplementary Figure S1).

### Cartilage and Bone Formation

**FIGURE 3:**
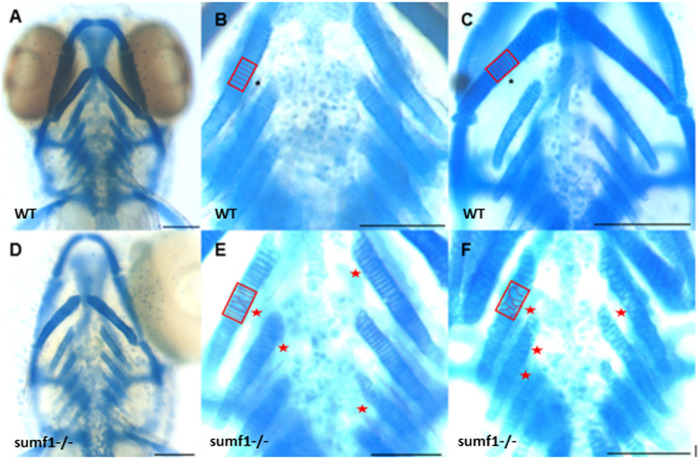
Intercalation of chondrocytes in the craniofacial skeleton. Alcian blue stained samples of **(A,B)** wildtype (*n* = 4) and **(D,E)** *sumf1* ^*−/−*^ zebrafish (*n* = 6) at 5 d.p.f. The chondrocytes of wildtype larvae **(B)** at 5 d.p.f. were neatly stacked (asterisk) indicating complete intercalation whereas intercalation in *sumf1* ^*−/−*^ larvae **(E)** was disrupted in some areas (outlined) and stacked elsewhere (red star). **(C,F)**. At 10 d.p.f. (C&F, *n* = 3 per genotype), the intercalation of chondrocytes was disrupted in **(F)** *sumf1* ^*−/−*^ larvae (asterisk and red box) when compared to **(C)** wildtype (asterisk). Scalebar represents 200 μm.

**FIGURE 4:**
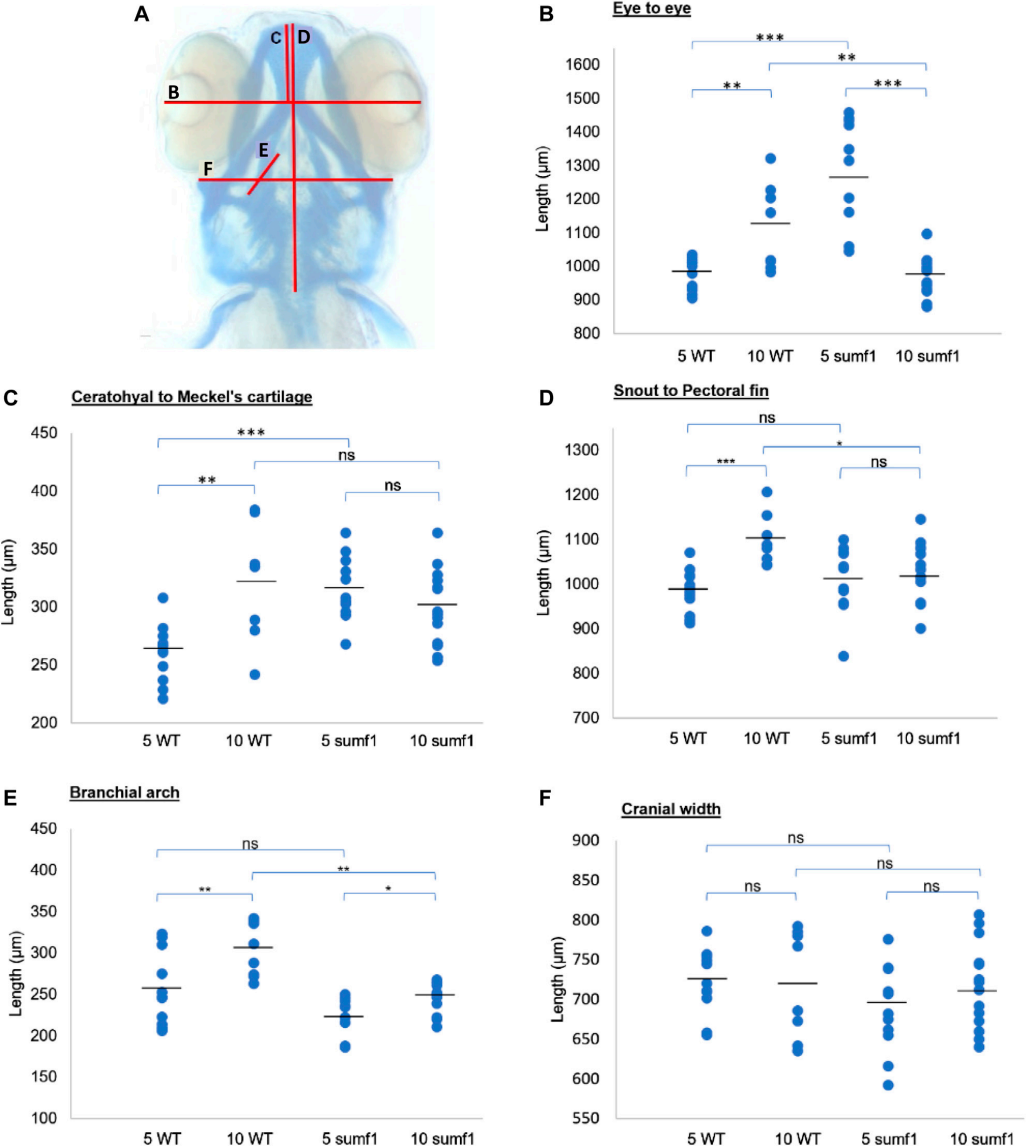
Cranial cartilage measurements of wildtype and *sumf1* mutant zebrafish at 5 and 10 d.p.f. **(A)** Schematic diagram indicating measurements of cartilage element representing the following: The distance between the eyes **(B)**, Meckel’s cartilage and ceratohyal **(C)**, snout and the pectoral fin **(D)**, the length of the first branchial arch **(E)**, cranial width **(F)**. Differences in the mean of each measurement was analysed using Welch’s t-test (wildtype and *sumf1* ^*−/−*^; 5 d.p.f. and 10 d.p.f.) and the *p*-values are denoted as following: ns: *p* > 0.05, *: *p* ≤ 0.05, **: *p* ≤ 0.01, and ***: *p* ≤ 0.001. Means values are indicated by the line.

These finding were surprising since the mouse *Sumf1* ^*−/−*^ model and human MSD patients with null mutations often suffer from early mortality and growth retardation. We therefore looked in detail at the development of *sumf1* ^*−/−*^ embryos and larvae to determine whether they displayed any of the morphological defects observed in *Sumf1* ^*−/−*^ knockout mouse model and human MSD patients. Analysis of craniofacial cartilage development revealed a defect in the intercalation of chondrocytes in the branchial arches in larvae at 5 d.p.f. and this persisted at 10 d.p.f. (Figures 3A–F). In addition, we measured a range of craniofacial dimensions to determine whether *sumf1* ^*−/−*^ larvae displayed the same widening of the head and growth retardation as observed in mouse models and human MSD patients (Figure 4A). Analysis of larvae at 5 and 10 d.p.f. revealed differences in measurements between wildtype and *sumf1* ^*−/−*^ larvae at these timepoints, as well as differences in the growth of these elements between the two groups (Figures 4B–F). The greatest difference observed between wildtype and *sumf1* ^*−/−*^ larvae at 5 d.p.f. was the eye-to-eye width (Figure 4B) and length from the ceratohyal to Meckel’s cartilage (Figure 4C). These measurements indicate that the cranial shape of this region was narrower and shorter in *sumf1* ^*−/−*^ larvae at 5 d.p.f. compared to wildtype, which is consistent with the cranial deformation seen in MSD patients and mouse models. In addition, these two measurements, as well as the snout to pectoral fin length (Figure 4D) increased significantly in wildtype larvae between 5 and 10 d.p.f. (indicative of growth/elongation of the head), whereas no growth was observed in *sumf1* ^*−/−*^ larvae suggesting that development and growth of the facial cartilages of *sumf1* ^*−/−*^ zebrafish was retarded compared to wildtype larvae. The change in eye-to-eye width in *sumf1* ^*−/−*^ larvae between 5 and 10 d.p.f. was surprising and is likely to result from a mechanism different to cartilage development since it resolves over time (e.g., interim oedema), as one would not expect a reduction in this measurement during development unless it were coupled with growth in perpendicular axis (e.g., ceratohyal to Meckel’s cartilage or ceratohyal to Meckel’s cartilage).

**FIGURE 5:**
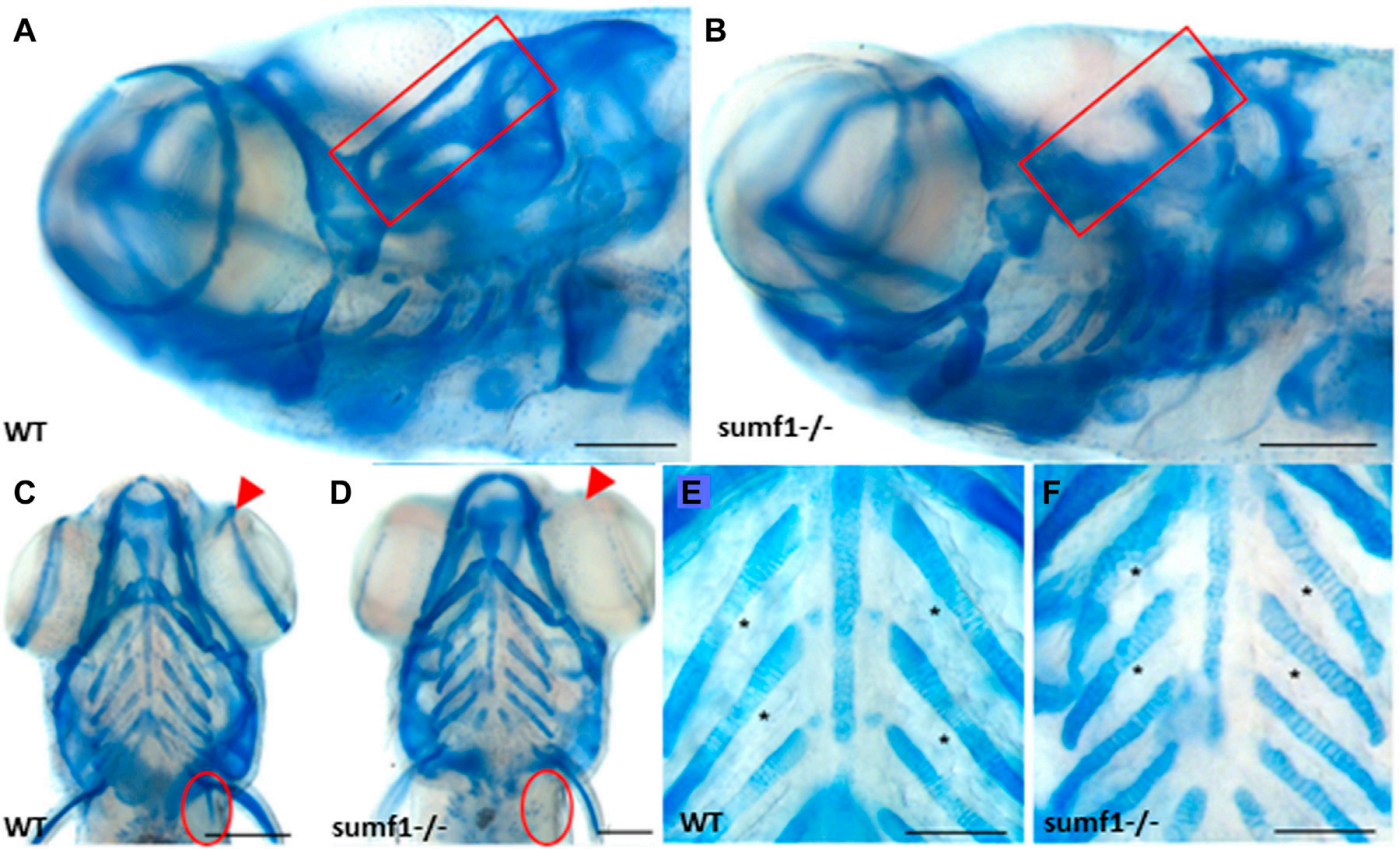
Analysis of craniofacial cartilage at 30 d.p.f. The semi-circular canal (red box) and the scapulocorocoid (red circle) were present in wildtype **(A,C)** were missing in *sumf1* ^*−/−*^ larvae **(B,D)**. Similarly, supraorbital cartilage (red arrow) was well developed in wildtype larvae **(C)** but retarded in *sumf1* ^*−/−*^ larvae. Notably, chondrocytes in the branchial arches were neatly stacked (intercalated) in wildtype larvae **(E)**, whereas the intercalation of the chondrocytes was disrupted in *sumf1* ^*−/−*^ larvae **(F)**. Scalebar represents 200 μm. Representative images of *n* = 3 samples for each genotype.

We next explored whether the defects in the cartilaginous elements of the craniofacial skeleton persisted into adulthood and whether defects in bone formation were observed in zebrafish larvae at 10, 15, and 30 d.p.f. The most striking differences in the cartilaginous skeleton at 30 d.p.f. was the lack of specific cartilaginous structures in *sumf1* ^*−/−*^ zebrafish, namely the otic capsule (*n* = 3; 67%) (Figures 5A,B) and the scapulocorocoid, and reduction of staining in the supraorbital cartilage, indicative of retarded formation (Figures 5C,D). The intercalation of chondrocytes into the branchial arch elements in wildtype fish remained uniform, with narrow cells appearing neatly stacked (Figure 5E). In contrast, in *sumf1* ^*−/−*^ zebrafish, the chondrocytes showed some degree of stacking but were not as tightly stacked as seen in wildtype (Figure 5F) leading to distortion (bending) of the branchial arches due to the irregular round cell shape.

**FIGURE 6:**
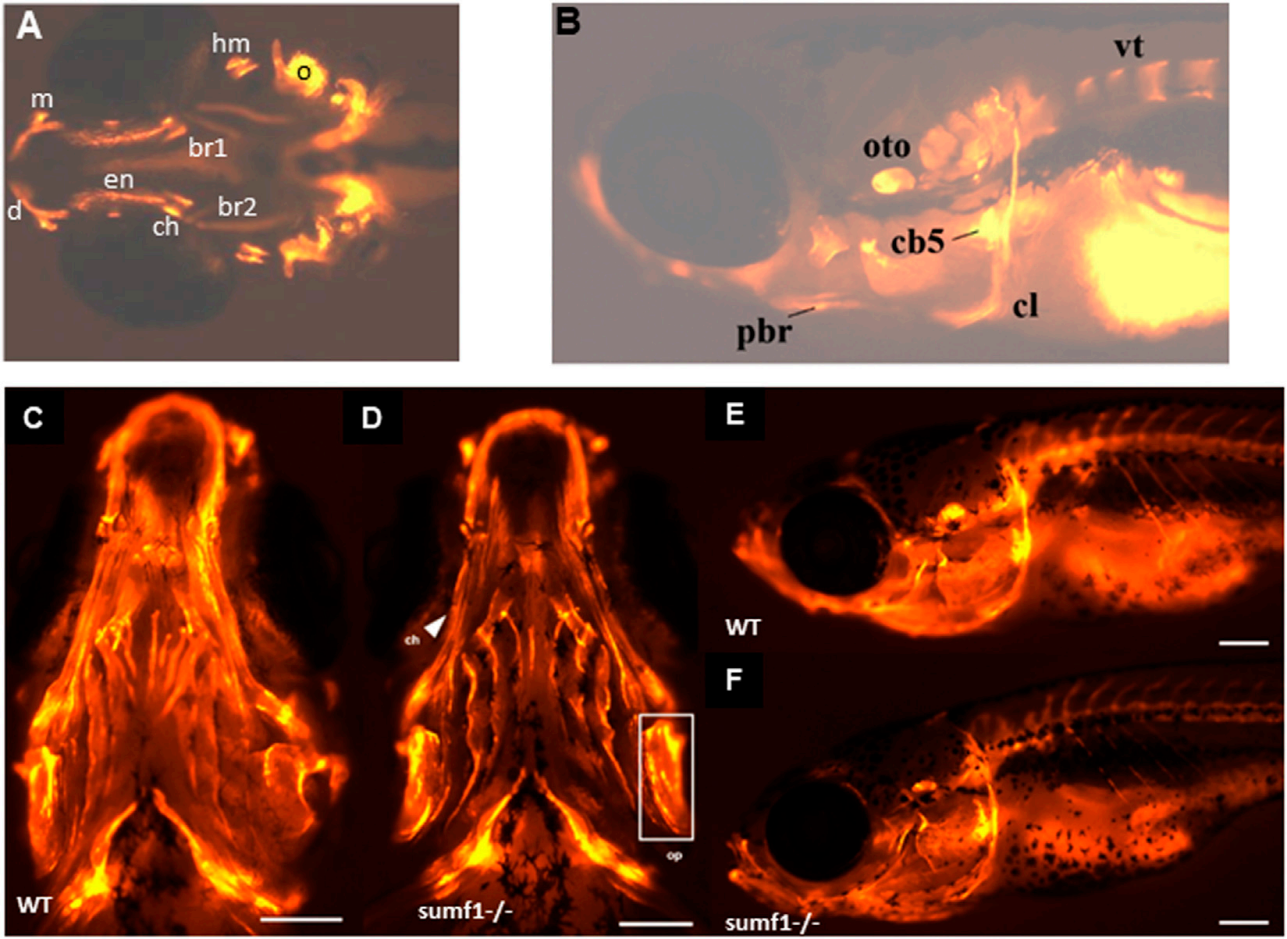
Development of ossification centres in different craniofacial elements. **(A,B)** Alizarin red stained larvae were imaged at 10 and 15 d.p.f. and scored according to the size of the ossification centre (growth) and intensity of the fluorescent stain. Ossification centres in the vertebrae (vt), otoliths (oto), primary ossification center (oc) for the hyomandibular (hyo), and ceratohyal (ct), maxilla (m), dentary (d), cleithrum (cl), posterior branchiostegal ray (pbr), ceratobranchial 5 (cb5), operculum (op), entopterygoid (en), branchiostegal ray (bcr) 1 and 2 were assessed (see Table 2). At 30 d.p.f., no overt differences in the mineralisation/ossification of the craniofacial skeleton could be identified between wildtype **(C,E)** and *sumf1* ^*−/−*^ larvae (D&F). No differences were found in the fluorescent intensity of the operculum (white box) and ceratohyal (white arrow). The scalebar represents 500 μm. Figure shows representative images of *n* = 3 samples for each genotype.

In zebrafish, ossification of endochondral bone in the craniofacial region begins at 3 d.p.f. but occurs at different timepoints in different elements and it is only from 14 d.p.f. that all ossification centres are present (Cubbage and Mabee, 1996). To examine whether these changes in the cartilage of the endochondral skeleton affected ossification, we performed *in vivo* bone staining using Alizarin red. The ossification centres of the different craniofacial elements (Figures 6A,B) were analysed at 10 and 15 d.p.f. and scored according to the size of the ossification centre (growth) and intensity of the fluorescent stain (degree of mineralisation), using a 4-point scoring system, for 13 different ossification centres. The results for each ossification centre are summarised in Table 2 with the mean score for all elements. At 10 d.p.f., *sumf1* ^*−/−*^ zebrafish showed a significant reduction in mean score of all the ossification centres (*p* < 0.05, Welch’s t-test) when compared to wildtype. However, when looking at the ossification of individual elements, only the otoliths were significantly more developed (*p* < 0.05, Welch’s t-test) in wildtype, compared to *sumf1* ^*−/−*^ zebrafish, indicating that the mean score represents cumulative modest changes in ossification between wildtype and *sumf1* ^*−/−*^ larvae.

**TABLE 2:** Quantification of ossification centres in different craniofacial elements.

Treatment	vt	Oto	Oc (hyo)	Oc (ct)	m	d	cl	pbr	Cb5	op	en	Br 1	Br 2	Total (mean)
Day 10 WT (*n* = 6)	*	**	*	*	**	**	**	**	**	**	**	*	*	13.67
Day 10 sumf1 (*n* = 9)	*	*	*	*	**	*	*	*	*	*	*	*	*	10.11
Day 15 WT (*n* = 7)	***	***	****	****	***	****	***	***	***	****	****	****	****	39.29
Day 15 sumf1 (*n* = 5)	**	**	*	*	***	**	***	***	***	***	**	**	**	22.40

The ossification centres of the different craniofacial elements (Figure 6A) were analysed at 10 and 15 d.p.f. and scored according to the size of the ossification centre (growth) and intensity of the fluorescent stain (degree of mineralisation), using a 4-point scoring system, for 13 different ossification centres. Ossification centres in the vertebrae (vt), otoliths (oto), primary ossification center (oc) for the hyomandibular (hyo) and ceratohyal (ct), maxilla (m), dentary (d), cleithrum (cl), posterior branchiostegal ray (pbr), ceratobranchial 5 (cb5), operculum (op), entopterygoid (en), branchiostegal ray (bcr) 1 and 2 were assessed. The mean scores for each element were summarised as *≤1, **≤2, ***≤3, ****≤4.

Similarly, the mean ossification score of *sumf1* ^*−/−*^ was also reduced at 15 d.p.f. (*p* < 0.05, Welch’s t-test), with significantly less development of both the hyomandibular and the ceratohyal primary ossification centers (*p* < 0.05, Welch’s t-test) in *sumf1* ^*−/−*^ compared to wildtype zebrafish. Furthermore, ossification of non-endochondral elements (i.e., dermal bones), such as the branchiostegal rays, were also significantly less developed in the *sumf1* ^*−/−*^ zebrafish. While these observations suggested that the retardation in bone development was still present at 15 d.p.f., the variation in different elements suggests that the effect of *sumf1* ^*−/−*^ null mutation on bone development was not uniform with longer bone elements, like the branchiostegal rays and entopterygoid significantly affected (*p* < 0.05, Welch’s t-test) at 15 d.p.f., whereas wider bone elements like operculum and ceratobranchial 5 were not significantly different (*p* > 0.05, Welch’s t-test).

Since these data suggested that cartilage deformities do translate into retarded bone ossification in *sumf1* ^*−/−*^ zebrafish, we next examined bone formation at 30 d.p.f. No overt differences were observed in qualitative comparisons of the Alizarin red staining between wildtype and *sumf1* ^*−/−*^ fish in any of the structures in terms of bone shape and intensity of fluorescent staining (Figures 6C–F). To support this observation, quantification of fluorescence intensity was performed on two bones; the ceratohyal (long bone element) and operculum (wide bone element). No significant differences were observed between wildtype and *sumf1* ^*−/−*^ fish, suggesting that the early defects in cartilage formation did not persist into adulthood.

### Immune Cell Responses

**FIGURE 7:**
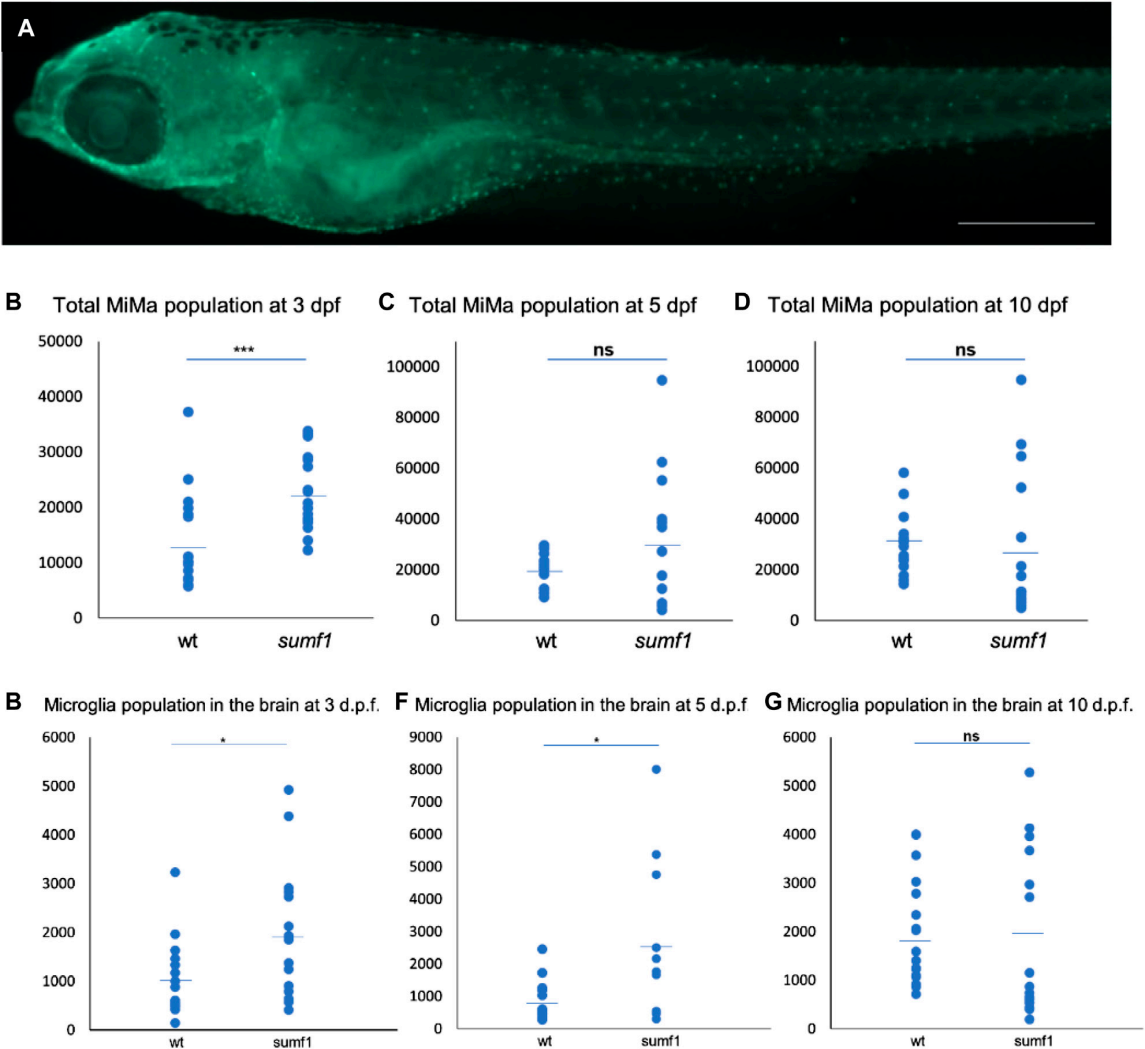
Quantification and distribution of microglia and macrophages. **(A)** Wildtype and *sumf1* ^*−/−*^ larvae were immunostained with an antibody to detect microglia and macrophages. Scalebar represents 200 μm. The total number of microglia and macrophages (Mi/Ma) was quantified at 3, 5, and 10 d.p.f. by analysing maximum intensity projections of a Z-stack through the entire larva. **(B)** At 3 d.p.f., there was a significant increase in the Mi/Ma population in *sumf1* ^*−/−*^ larvae compared to wildtype larvae. However, these differences did not persist at **(C)** 5 d.p.f. and **(D)** 10 d.p.f. **(E–G)** The brain resident population of labelled cells (assumed to by microglia) was quantified at 3, 5, and 10 d.p.f. The brain was selectively quantified by selecting the same Region of Interest (ROI) targeting the frontal and the dorsal brain in all images. There was a significant increase in the microglia population in *sumf1* ^*−/−*^ larvae at **(E)** 3 d.p.f. and **(F)** 5 d.p.f. but no difference at 10 d.p.f. **(G)**. Two-tailed t-test with *p*-values are denoted as follows: ns: *p* > 0.05, *: *p* ≤ 0.05, **: *p* ≤ 0.01, and ***: *p* ≤ 0.001. Means values are indicated by the line.

One of the most evident features of pathology in the *Sumf1−/−* mouse model was the presence of highly vacuolated macrophages and activated microglia (Settembre et al., 2007). Indeed, activated microglia appear to contribute to neurodegeneration in mouse models of many lysosomal storage diseases (Bellettato and Scarpa, 2010). Based on these observations in mammalian models, we examined the number and distribution of microglia (Mi) and macrophages (Ma) (hereafter referred to as total Mi/Ma population) in our *sumf1* ^*−/−*^ zebrafish model (Figure 7A) at different time points. Quantification of wholemount antibody staining demonstrated that the total Mi/Ma population at 3 d.p.f. was significantly higher (*p* < 0.05, Welch’s t-test) in *sumf1* ^*−/−*^ than wildtype zebrafish (Figure 7B). However, no significant difference was observed at 5 and 10 d.p.f. (Figures 7C, D) suggesting that the expansion of the population observed at 3 d.p.f. does not persist. To investigate the growth in the total MiMa population throughout the 10 days, we compared the population size between consecutive days (3–5 d.p.f. and 5–10 d.p.f.) for each genotype. The Mi/Ma population increased in wildtype larvae from 3–10 d.p.f. (*p* < 0.05, Welch’s t-test), whereas this population, whilst initially higher in *sumf1* ^*−/−*^ zebrafish, did not increase significantly (*p* > 0.05, Welch’s t-test) over that same duration.

To investigate the possibility that the brain resident microglia population changes in a different manner to the total population, we selectively counted the microglia within the brain of the zebrafish (Figures 7E–G). A significant increase (*p* < 0.05, Welch’s t-test) in microglia in *sumf1* ^*−/−*^ larvae was observed at 3 d.p.f. (Figure 7E). and persisted at 5 d.p.f. (Figure 7F) but became similar to that of wildtype larvae at 10 d.p.f. (Figure 7G). Taken together, these data suggest that early differences in microglial and macrophage populations normalise over time, which was consistent with the cartilage and bone malformation data described earlier.

### Lysosome Characterisation and Autophagy

**FIGURE 8:**
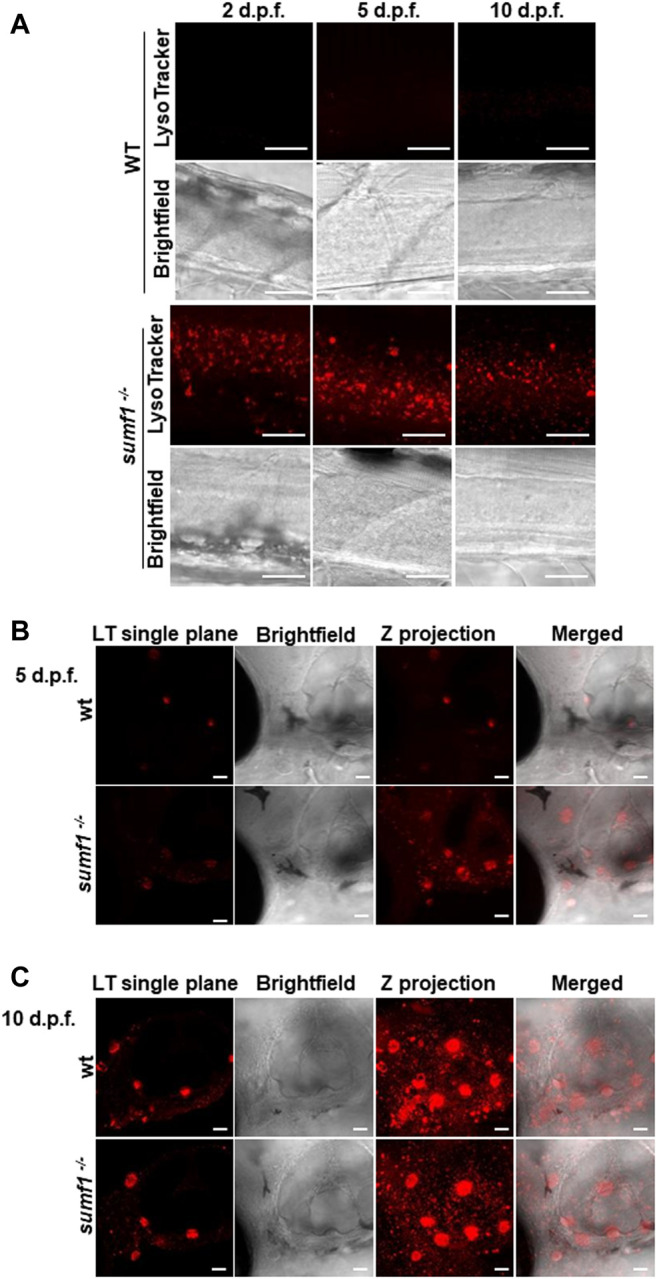
Imaging of lysosomes. *In vivo* lysotracker staining was imaged using confocal microscopy. **(A)** A striking increase in lysotracker staining was observed in the spinal cord of *sumf1* ^*−/−*^ larvae compared to wildtype (wt) larvae at 2 d.p.f., 5 d.p.f., and 10 d.p.f. **(B)** An increase in lysotracker staining was observed in and around the otic vesicle of *sumf1* ^*−/−*^ larvae compared to wt larvae at 5 d.p.f., whereas no differences between the two groups were observed at 10 d.p.f. **(A–C)** Representative images of the lysotracker staining, brightfield and the z projection. At least 5 fish were analysed for each group. Scale bar represents 30 µm for all images.

**FIGURE 9:**
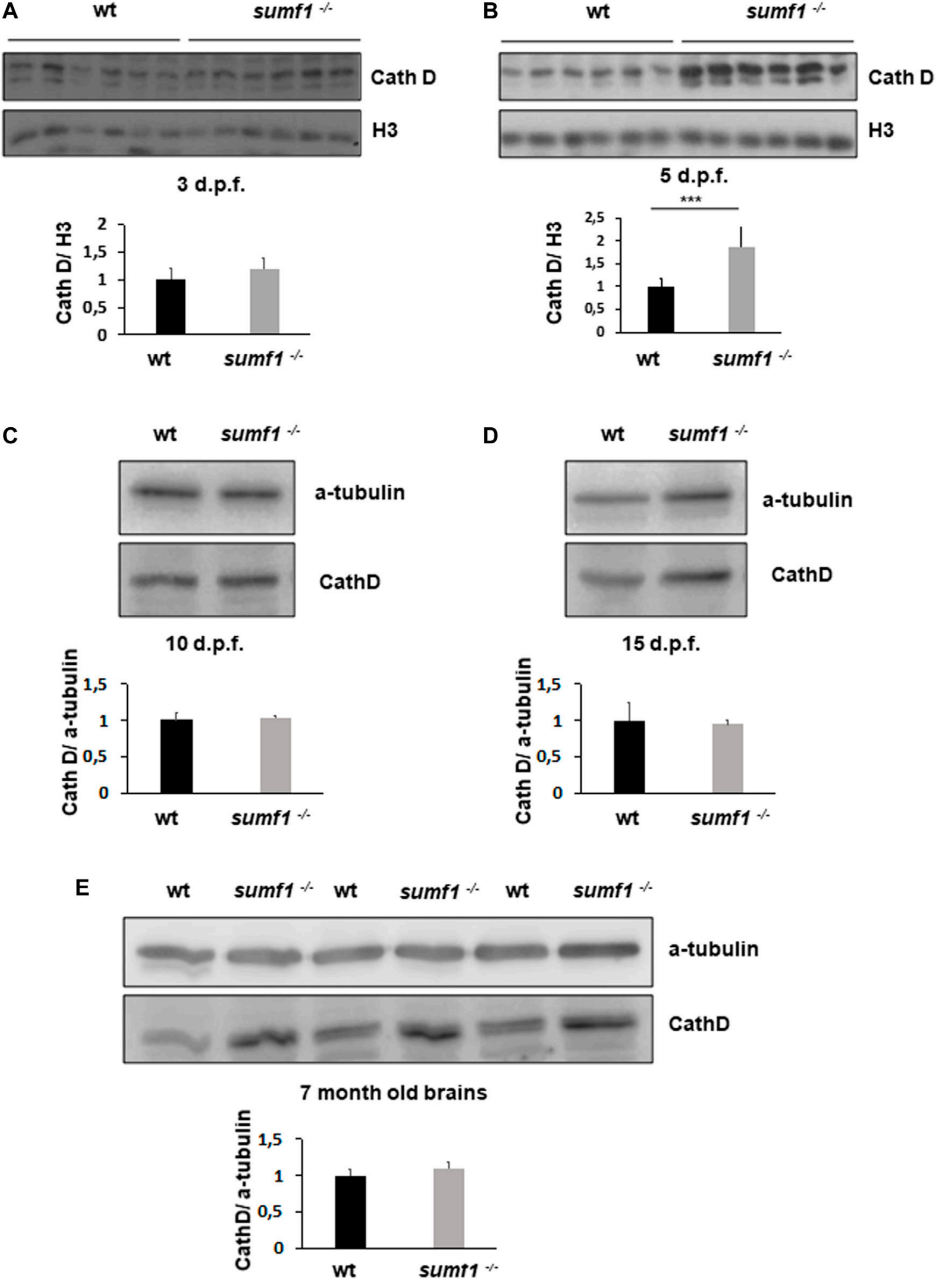
Cathepsin D protein levels as an indicator of lysosome digestive capacity. Western blotting was performed in larvae of different ages (3, 5, 10, and 15 d.p.f.) and on brains of adult zebrafish to measure the levels of the mature form of cathepsin D (37 kDa band presented) as an indicator of the digestive capacity of lysosomes. **(A)** No differences in cathepsin D were observed between wildtype (wt) and *sumf1* ^*−/−*^ fish at 3 d.p.f. The levels of cathepsin D are significantly increased at 5 d.p.f. in the *sumf1* ^*−/−*^ fish relative to wt fish **(B)** whereas no differences were observed between the different genotypes at 10 d.p.f. **(C)**, 15 d.p.f. **(D)** and in the brains of adult fish (7 months old), **(D)**. Graphs show mean values (± SEM) of densitometry of cathepsin D normalised to histone 3 (loading control at 3 d.p.f and 5 d.p.f.) and α-tubulin (loading control at 10 d.p.f. and 15 d.p.f. larvae and 7 months old brain) from at least 3 independent experiments. Statistical analysis was performed using a two-tailed t-test. ***p < 0.001.

In humans, MSD results in the accumulation of undigested GAGs within the lysosome which is associated with a lysosomal storage deficiency. Therefore, we assessed abundance and activity of lysosomes over a range of ages in our zebrafish *sumf1* ^*−/−*^ model. Lysotracker imaging of larvae from 2–10 d.p.f. revealed a marked increase in staining at all larval ages examined (Figure 8). We analysed staining in the spinal cord region (Figure 8A), since lysosome accumulation is known to occur in the CNS in other models and around the otic capsule (Figure 8B) because this region showed defects in cartilage and bone formation. The increase in Lysotracker staining may reflect an increase in lysosome numbers or increases in their size. Therefore, we examined cathepsin D levels as a marker of lysosome digestive capacity and as a surrogate marker for lysosome abundance. We observed a significant increase in cathepsin D levels in *sumf1* ^*−/−*^ larvae relative to wildtype controls at 3 and 5 d.p.f. (Figures 9A,B) but no difference at later larval ages (10 and 15 d.p.f., Figures 9C,D) nor in the brains of adult *sumf1* ^*−/−*^ fish. Interestingly, these increases in cathepsin D expression coincide with the earliest onset of defects that we noted in craniofacial development and increases in the Mi/Ma population in *sumf1* ^*−/−*^ larvae and the normalisation of cathepsin D levels correlates with recovery of the early morphological and cell population defects.

**FIGURE 10:**
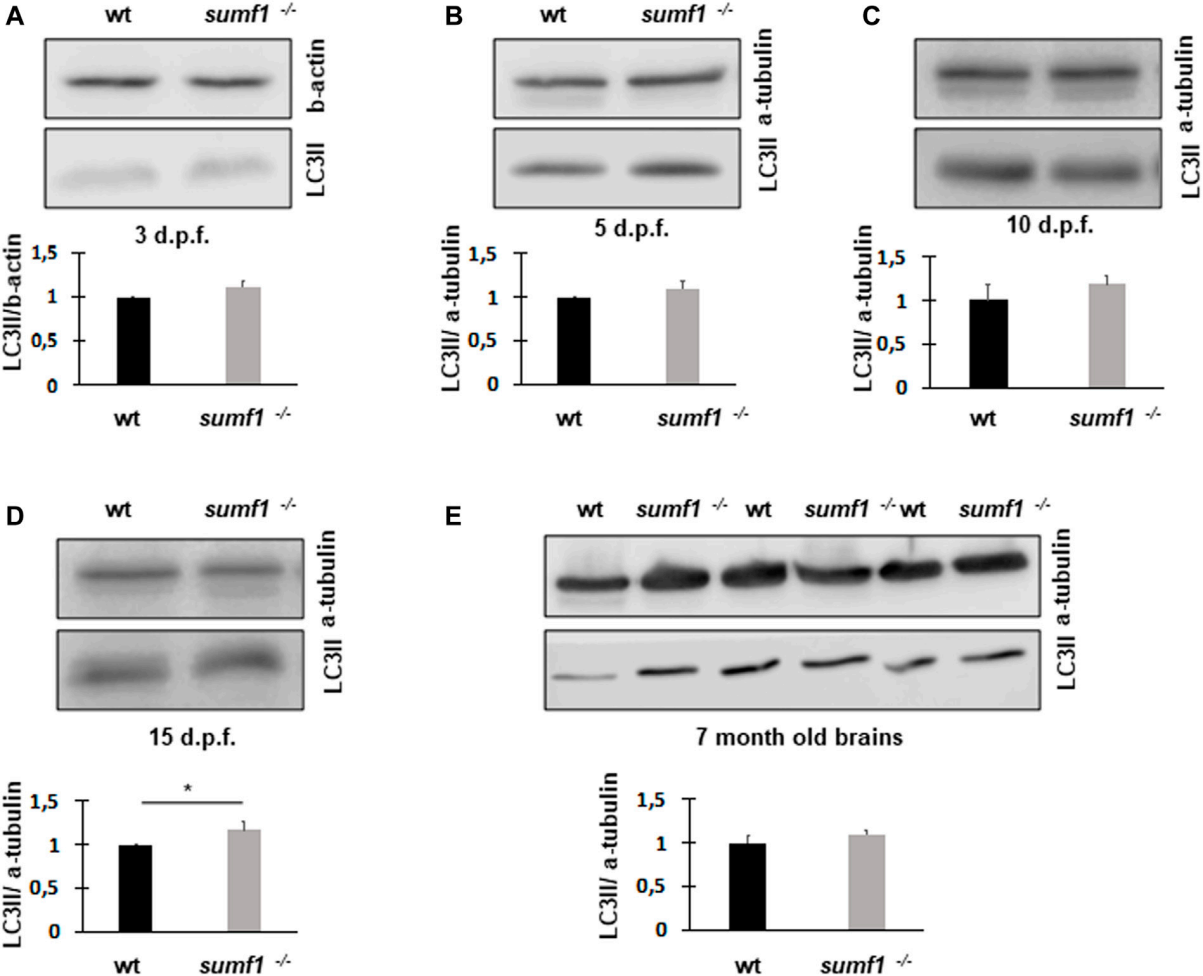
LC3-II protein levels as an indicator of autophagosome formation and flux. Western blotting was performed in larvae of different ages (3, 5, 10, and 15 d.p.f.) and on brains of adult zebrafish to measure the levels of LC3-II as a measure of autophagosome number. No differences in LC3-II levels were observed between wildtype (wt) and *sumf1* ^*−/−*^ fish at 3 d.p.f., 5 d. p.f., 10 d.p.f. Although a modest increase (1.28x) increase in LC3-II was observed at 15 d.p.f. this difference was not evident in the brains of adult fish (7 months). Graphs show mean values (±SEM) of densitometry of LC3-II normalised to β-actin (loading control at 3 d.p.f) and α-tubulin (loading control at 5 d.p.f., 10 d.p.f., 15 d.p.f. and 7 months) from at least 3 independent experiments. Statistical analysis was performed using a two-tailed t-test. *p < 0.05.

Next, we measured LC3-II levels as a marker of autophagy, since this degradative pathway is known to be compromised in lysosomal storage diseases. Since LC3-II levels correlate with the numbers of autophagosomes and autolysosomes, one would expect the marker to increase in lysosome storage disorders where autophagosomes cannot be degraded by dysfunctional lysosomes. Surprisingly, we found no changes in LC3-II levels between wildtype and *sumf1* ^*−/−*^ larvae at 3, 5, and 10 d.p.f. (Figures 10A–C). and only a modest (1.28 x increase) in LC3-II at 15 d.p.f (Figure 10D). In addition, LC3-II levels in the brains of adult (7 month old fish) were also not affected (Figure 10E).

**FIGURE 11:**
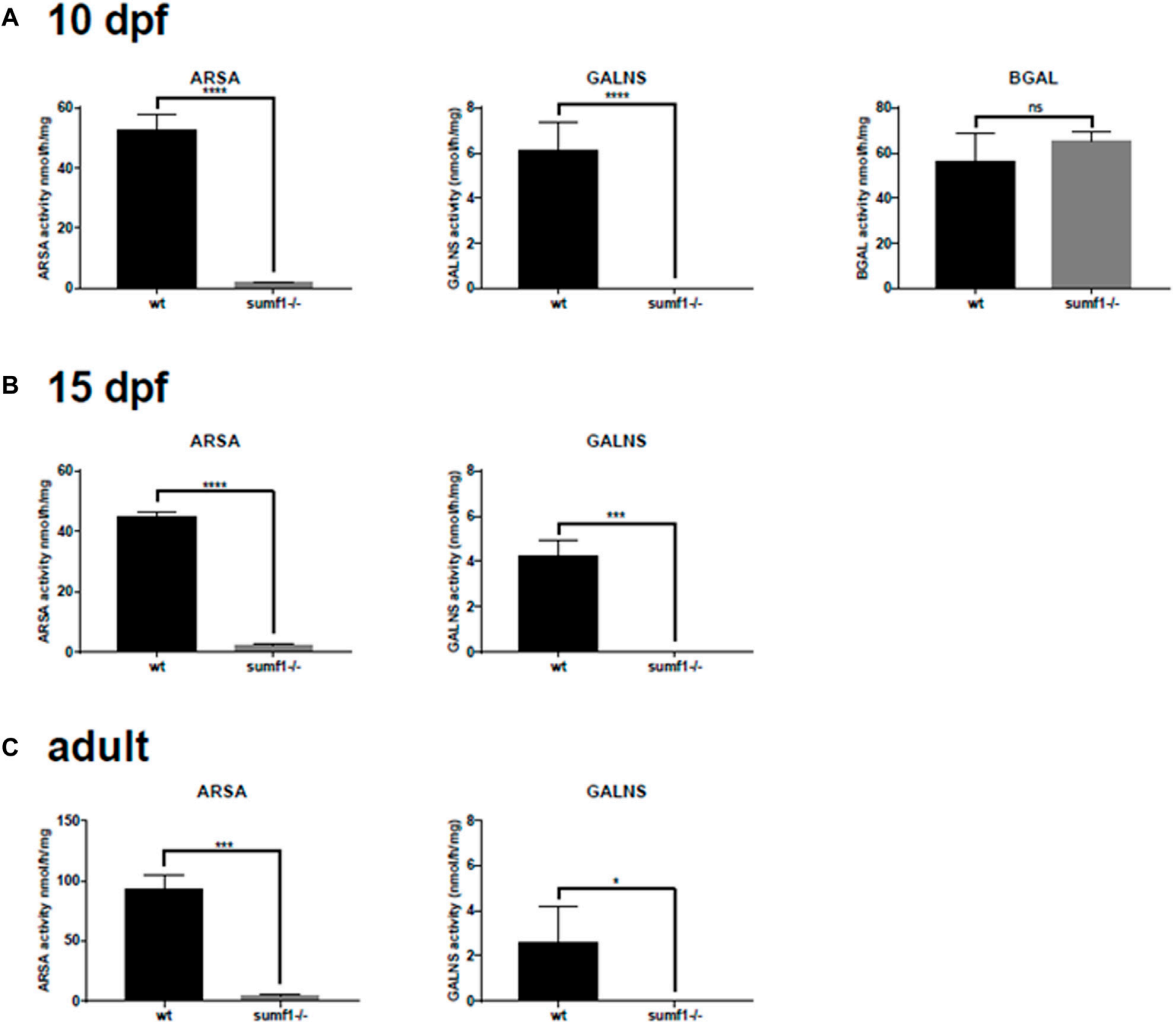
Sulfatase activity in older larvae and adult brains. Larvae at 5 d.p.f. showed no or neglible sulfatase activity (Figure 2B). However, since pathological phenotypes in *sumf1* ^*−/−*^ larvae resolved with age and adult *sumf1* ^*−/−*^fish were viable, we measured sulfatase activity at older timepoints. No or negligible levels of ARSA and GALNS activity could be detected in *sumf1* ^*−/−*^ larvae at 10 and 15 d.p.f., and in the brains of adult fish. Beta-galactosidase was included as a control as this enzyme is a lysosomal hydrolase which is not dependent on FGE. Graphs show mean values (±SD) from at least 3 biological replicates. Statistical analysis was performed using a two-tailed t-test. *p* > 0.05, *: *p* ≤ 0.05, **: *p* ≤ 0.01, and ***: *p* ≤ 0.001.

We postulated that the recovery in pathological phenotypes observed in *sumf1* ^*−/−*^ fish could be caused by two alternative mechanisms. Firstly, it may be caused by a recovery of sulfatase activity. Alternatively, it may be caused by the upregulation of unknown pathways that allow zebrafish to tolerate high levels of substrate accumulation or pathways that allow the degradation or removal of such substrates. To investigate this, we measured sulfatase activity in older larvae, at times when pathological phenotypes resolve, and in the brains of adult fish. In all ages investigated, no or negligible levels of ARSA and GALNS activity could be detected in *sumf1* ^*−/−*^ fish (Figures 11A–C), suggesting an absence of FGE activity with no change in Beta-galactosidase (used as a control).

## Discussion

The aim of our work was to characterise a zebrafish *sumf1* ^*−/−*^ null mutant with the expectation that this would recapitulate the severe features of MSD pathology seen in mouse and *Drosophila* models, notably early lethality, but with the benefit of being a vertebrate model organism that was amenable to high-throughput chemical and genetic screens, to identify targets for therapeutic intervention. Whilst young *sumf1* ^*−/−*^ larvae displayed some aspects of MSD pathology, namely facial dysmorphia, elevated microglia population and growth retardation, the recovery of these defects, and the survival of *sumf1* ^*−/−*^ to adulthood was unexpected. This, coupled with the normalisation of cathepsin D levels and no evidence of a block in autophagic flux suggests that the *sumf1* ^*−/−*^ zebrafish have compensatory mechanisms that allow normal lysosomal function in the absence of enzymes that, in all other organisms, are required to degrade GAGs and sulfolipids. This demonstrates that it is possible for a vertebrate organism to be viable and healthy in the absence of FGE function and when all cellular sulfatases are inactive. Our findings suggest that zebrafish possess a mechanism to circumvent the accumulation of lysosomal substrates that result in severe lysosomal disorders in mammals, perhaps by compensating for dysregulated intracellular pathways and lipid membrane turnover, features that are characteristics of lysosomal disorders in non-vertebrates (Platt et al., 2012). Despite the fact that this model is not suitable for viability screens, it offers the potential for identifying alternative intracellular signalling pathways which, if existing and targeted in man, could be therapeutic strategies for the treatment of MSD and possibly other LSDs.

## Data Availability

The original contributions presented in the study are included in the article/Supplementary Material, further inquiries can be directed to the corresponding authors.
