# Supplementary material for: Unexpected Phenotype Reversion and Survival in a Zebrafish Model of Multiple Sulfatase Deficiency
Source: Front Cell Dev Biol. 2022 Jun 2;10:843079. doi: 10.3389/fcell.2022.843079 (PMC9203071; doi:10.3389/fcell.2022.843079)
Supplement: Supplementary file 1 [file DataSheet1.PDF]

Supplementary Figure 1

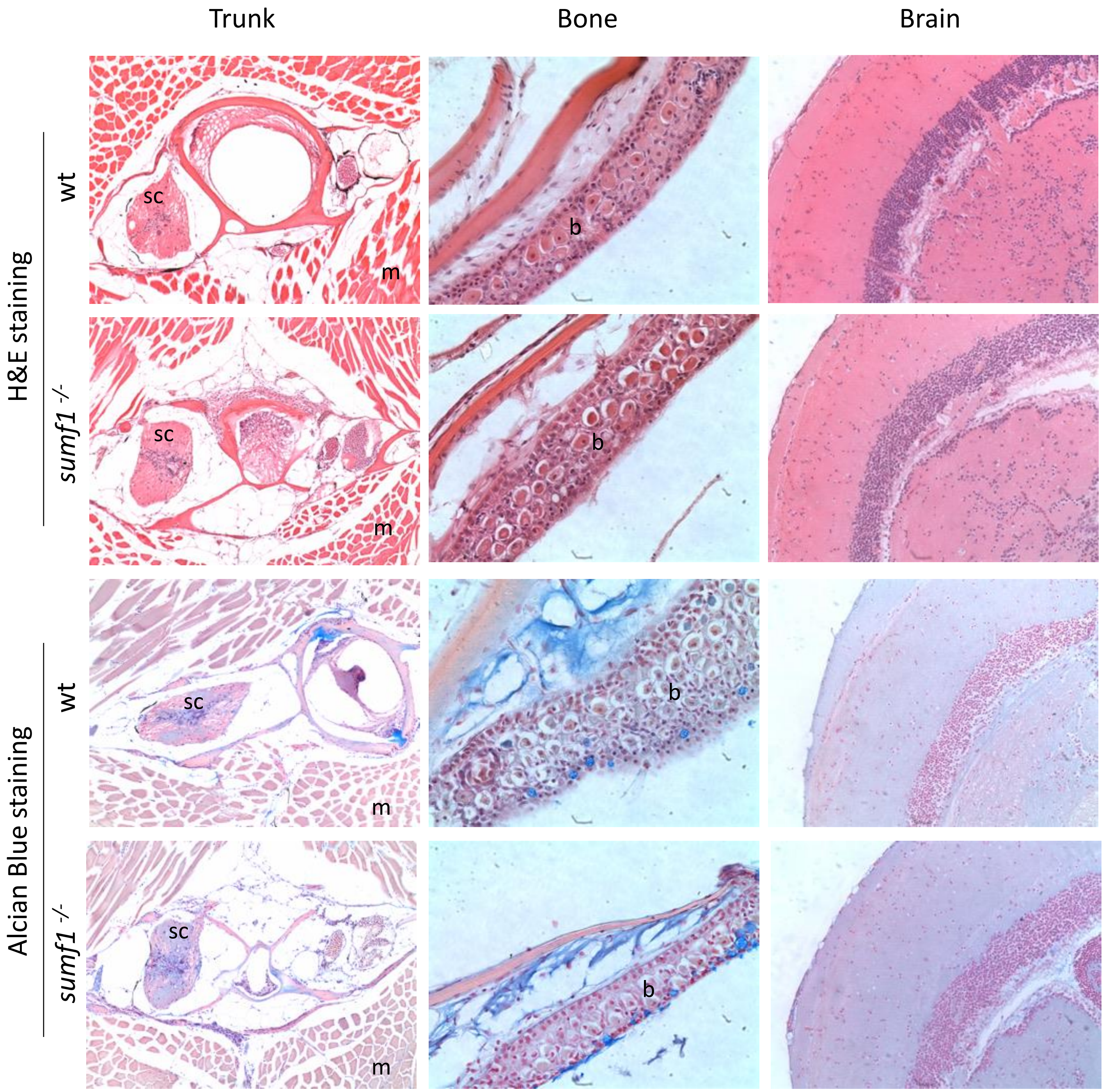

Supplementary figure 1: Histological analysis of wildtype and *sumf1*$^{-/-}$ adult tissues.
No overt differences were observed in histological sections of *sumf1*$^{-/-}$ fish at 12 months old. H&E staining (upper panels) was used to examine general features and Alcian Blue staining was used to visualize GAG accumulation (blue staining). Sections through the trunk show the spinal cord (sc) surrounded by vertebral arches and muscle (m). Trunk sections are orientated with dorsal to the left. An increase in Alcian Blue staining was observed in some regions of bone (b) but was not seen consistently in all samples.
